# Stakeholder perspectives on the current status and potential barriers of patient involvement in health technology assessment (HTA) across Europe

**DOI:** 10.1017/S0266462324004707

**Published:** 2024-12-19

**Authors:** Anke-Peggy Holtorf, Neil Bertelsen, Hannes Jarke, Maria Dutarte, Silvia Scalabrini, Valentina Strammiello

**Affiliations:** 1 Health Outcomes Strategies GmbH, Basel, Switzerland; 2 College of Pharmacy, University of Utah, Salt Lake City, UT, USA; 3 Patient and Citizen Involvement in HTA Interest Group, Steering Committee, Health Technology Assessment international (HTAi), Edmonton, AB, Canada; 4 Neil Bertelsen Consulting, Berlin, Germany; 5 European Patients’ Forum (EPF), Brussels, Belgium; 6 Centre for Business Research, Judge Business School, University of Cambridge, Cambridge, UK; 7 The European Patients’ Academy on Therapeutic Innovation (EUPATI), Utrecht, The Netherlands

**Keywords:** health technology assessment, patient participation, Europe, surveys and questionnaires, health services accessibility

## Abstract

**Background:**

There are wide variations in the practices of patient involvement in health technology assessment (HTA) in Europe. The field is lacking a consensus on good practices, leading to divergent processes, methods, and evaluation of patient involvement. To identify potential good practice approaches and current gaps, a structured online survey was conducted among HTA stakeholders, including HTA practitioners, patient stakeholders, industry representatives, and others who had experienced patient involvement in HTA.

**Methods:**

The questionnaire was co-created by HTA experts, patient stakeholders, and industry representatives and disseminated between 29 April and 14 September 2022.

**Results:**

Responses (n = 168) were submitted from thirty-two European countries by HTA practitioners (n = 33), patient stakeholders (n = 75), industry stakeholders (n = 42), providers (n = 5), academics (n = 7), and others (n = 6). The responses indicated that “*allowing access to treatments that have demonstrated value”*is the principle rationale for conducting HTA. In terms of the importance of patient involvement, there was consensus across stakeholder groups that “*patients have insights and information [that] no other stakeholder has*” and that patient involvement is important “*to inform HTA which evidence is most patient-relevant*”. Shortcomings were identified in the lack of systematic and transparent processes, an unsatisfactory level of information and guidance, and minimal communication and collaboration.

**Conclusions:**

The diverse stakeholders who responded highlighted the need for improving specific aspects of patient involvement practices, including better guidance and information, a more consistent flow of communication between the HTA body and participating patient stakeholders, and the need to develop and implement a consensus on good practices.

## Introduction

Involving patients is widely recognized as an essential attribute of health technology assessments (HTAs) (1). The rationale for patient involvement in HTA as a means to improve methods and meaningfulness of the HTA has been broadly documented, and HTA bodies across Europe and worldwide provide varying levels of related processes, guidance and support (1–4).

In 2014, the Patient and Citizen Involvement in HTA Interest Group (PCIG) of Health Technology Assessment international (HTAi, www.HTAi.org) released a set of values and quality standards for patient involvement in HTA resulting from a Delphi process with the HTA membership (5). These constitute a consensus framework for patient involvement including fundamental requirements for involving patients meaningfully in HTA processes. Herein, patient involvement encompasses both the direct input from patients into the HTA alongside research on patient needs, experiences, and preferences (2). Within this context, patient stakeholders considered may include patients, caregivers, patient organizations, or advocates.

Today, many HTA bodies across Europe involve patients to varying degrees using a variety of methods (2,6). However, there is no broad consensus yet on the best methods or processes to do this. Some HTA bodies have well-documented processes along the HTA lifecycle and dedicated, qualified personnel for supporting such patient involvement. Others are in the process of piloting approaches to patient involvement that match their current resources (7,8).

Variations in patient involvement procedures occur in the timelines of conducting an HTA, the time available to patients to give input, the depth of the involvement (consultation, written submission, participation, co-creation), or the support provided (e.g., resources, training, coaching) (6,9,10).

In the absence of a consensus on good practices, the PCIG identified a need to explore experiences of patient involvement practices across Europe. Unfortunately, most HTA agencies do not systematically evaluate their patient involvement processes (11) or do not publish the results from any internal evaluations.

Therefore, PCIG, in partnership with the European Patients’ Academy on Therapeutic Innovation (EUPATI) and the European Patients’ Forum (EPF), developed a structured survey instrument to be deployed to relevant stakeholders, including HTA practitioners, patient stakeholders, industry representatives, and others who had experienced patient involvement in HTA. This paper reports on the key findings related to the drivers of involvement, motivations, methods, support, and perceived barriers.

## Methods

### Survey

A questionnaire was developed using an eight-step methodology as described, hereafter.

One: A scoping literature review identified the key concepts of patient involvement and the gaps as reported in the peer-reviewed literature.

Two: An online multiperspective workshop was convened. Participants included two members of HTAi; two participants from EUPATI; two participants from EPF, and one participant each from two industry associations, the Pharmaceutical Research and Manufacturers of America (PhRMA) and the European Federation of Pharmaceutical Industries and Associations (EFPIA). Starting with the findings of the scoping literature review, participants in a collaborative structured prioritization exercise agreed on key domains and subdomains to be explored in the survey.

Three: A first version of the questionnaire was drafted in English.

Four: Representatives of HTAi, EUPATI, and EPF conducted an iterative review to reach an agreement on the questionnaire content.

Five: The questionnaire was programmed as an online survey using the open source LimeSurvey software hosted on a firewall-protected server based in Europe, where the responses were also collected.

Six: Three EUPATI fellows (patient stakeholders who have passed the EUPATI Patient Expert Training Program on medicines research and development) tested the survey for functionality and comprehensibility.

Seven: After further revision addressing the tester feedback, the survey was translated and programmed to allow respondees to chose between French, Spanish, German, Italian, and Polish language. Changes introduced between the first and the final version of the questionnaire included formatting, adaptations of question wording or response choice, and changes to the type of response (e.g., ranking instead of rating).

Eight: The survey link was promoted through the networks of EUPATI, EPF, and HTAi for data collection between 29 April and 14 September 2022. Invitations were also publicized through two webinars to members of the patient organizations and the participants in the 2022 HTAi Annual Meeting.

#### Questionnaire structure

**Figure 1.**
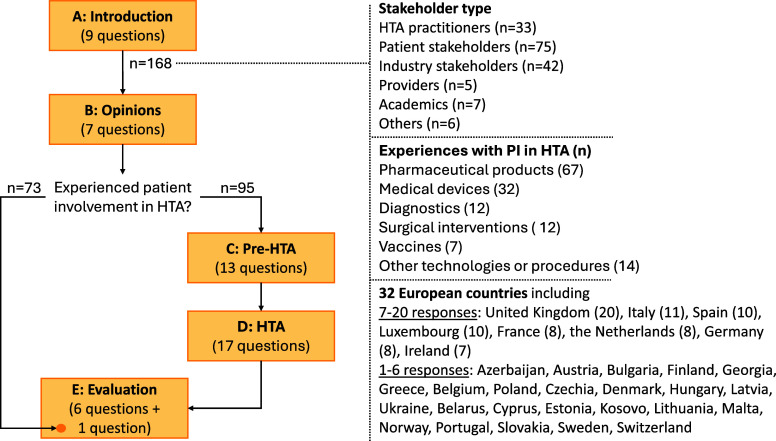
Survey flow chart with participant numbers and demographics.

The questionnaire is available as Supplementary Material S1. As depicted on the left side of Figure 1, it comprises five sections including (A) *Introduction* (nine questions) (B) *Opinions* (seven questions), (C) *Pre-HTA* (thirteen questions), (D) *HTA* (seventeen questions), and (E) *Evaluation* (seven questions). Not all participants received all of the questions; some questions were only visible for selected responder types (e.g., patients) or to those who had given a specific answer in a previous question. Questions in section C and D were only asked to those who confirmed having experienced an HTA with patient involvement. Those without such direct experience only received the questions in section A and B and the final question related to perceived barriers.

Except for the final question, all questions were in a multiple-choice form. Some offered an option for commenting or adding other choices. The final question invited the participants to identify up to five barriers they perceived for patient involvement.

After the closure of the survey, nonvalid responses (e.g., those without input or missing the responder country) were deleted. The valid responses were exported to a spreadsheet format (Microsoft Excel®) and analyzed by stakeholder groups (patients, HTA practitioners, others) as well as across all responders. Scores were calculated by weighting each vote according to the attributed rank (that is with four options; rank one = factor four, rank two = factor three, rank three = factor two, rank four = factor one) and calculating the average score for each option.

All open-text responses on barriers were collected and screened by one researcher for main themes (resulting in a first coding). A second researcher critically reviewed the first researcher’s coding of main themes to identify any discrepancies or gaps, which were then resolved in the discussion of the two researchers. Three independent reviewers (public health students) then coded all responses independently of the two researchers. To identify the level of agreement between raters, Fleiss’ Kappa was calculated to depict the inter-rater reliability between more than two raters (in this case, four). Codification by the independent reviewers showed agreement beyond chance for almost half of the 368 replies across all reviewers (Fleiss’ kappa = 0.447, 95 percent CI [0.437, 0.456]). Reviewers unanimously agreed on disputed codifications in a follow-up discussion.

## Results

### Demographics

A total of 168 responses related to thirty-two European countries (see right side of Figure 1). Most responders were HTA practitioners, patient stakeholders, or industry stakeholders. Among the other stakeholders who submitted responses were providers and academics. Ninety-five (56.6 percent) individuals had experienced patient involvement in HTA.

These experiences of patient involvement in HTA related to a variety of technologies with the majority being for pharmaceutical products, medical devices, diagnostics, or surgical interventions (see right side of Figure 1).

### Rationale for HTA and for patient involvement in HTA

Nine choices were provided for identifying the objectives of HTA. The three options: “*Allow access to treatments that have demonstrated value,”*, “*Understand the value of new treatments,”*, and “*Support evidence-based decisions on the access to treatments”* were ranked highest across all participants (see Table 1). When analyzing the ranking within each of the responder types (HTA, patients, industry), there appear to be slight differences; for example, HTA representatives ranked “*Understand the value of new treatments”* and “*Support evidence-based decisions”* higher than the other respondents. However, with the low number of HTA, industry or other respondents, these differences may not be meaningful. Despite the small sample sizes, exploratory quantitative analyses indicated that if there was a difference between stakeholders at all, then patient stakeholders may have been slightly more in favor of more patient involvement than other groups (R^2^ = 0.072; p = 0.001).

**Table 1.** Results for ranking possible reasons for performing HTA. The participants could rank a maximum of five responses. In total, 113 participants responded to the question including 25 HTA practitioners, 52 Patient stakeholders, 33 Industry stakeholders, and 3 others

Reason for HTA	All	HTA	Patients	Industry
*N=*	113*	*25*	*52*	*33*
Allow access to treatments that have demonstrated value	2.6	2.7	2.9	2.7
Understand the value of new treatments	2.4	1.0	2.5	3.3
Support evidence-based decisions on the access to treatments	2.3	2.8	2.3	1.8
Ensure evidence-based allocation of healthcare resources	1.9	2.5	1.8	1.8
Equity for users of healthcare products and services	1.3	1.0	1.4	1.4
Rationalize healthcare decisions	1.2	1.55	0.7	1.2
Ensure that treatments are affordable	1.1	0.35	1.1	1.5
Protect healthcare system from treatments that are less effective	1.0	1.39	1.0	0.9
Save cost in healthcare	0.4	0.32	0.3	0.5

### Barriers to patient involvement

It is often argued that it is difficult to find patients willing to give input to HTA (12). Hence, all participants were asked to rank the main reasons that prevent patients from being more involved.

**Figure 2.**
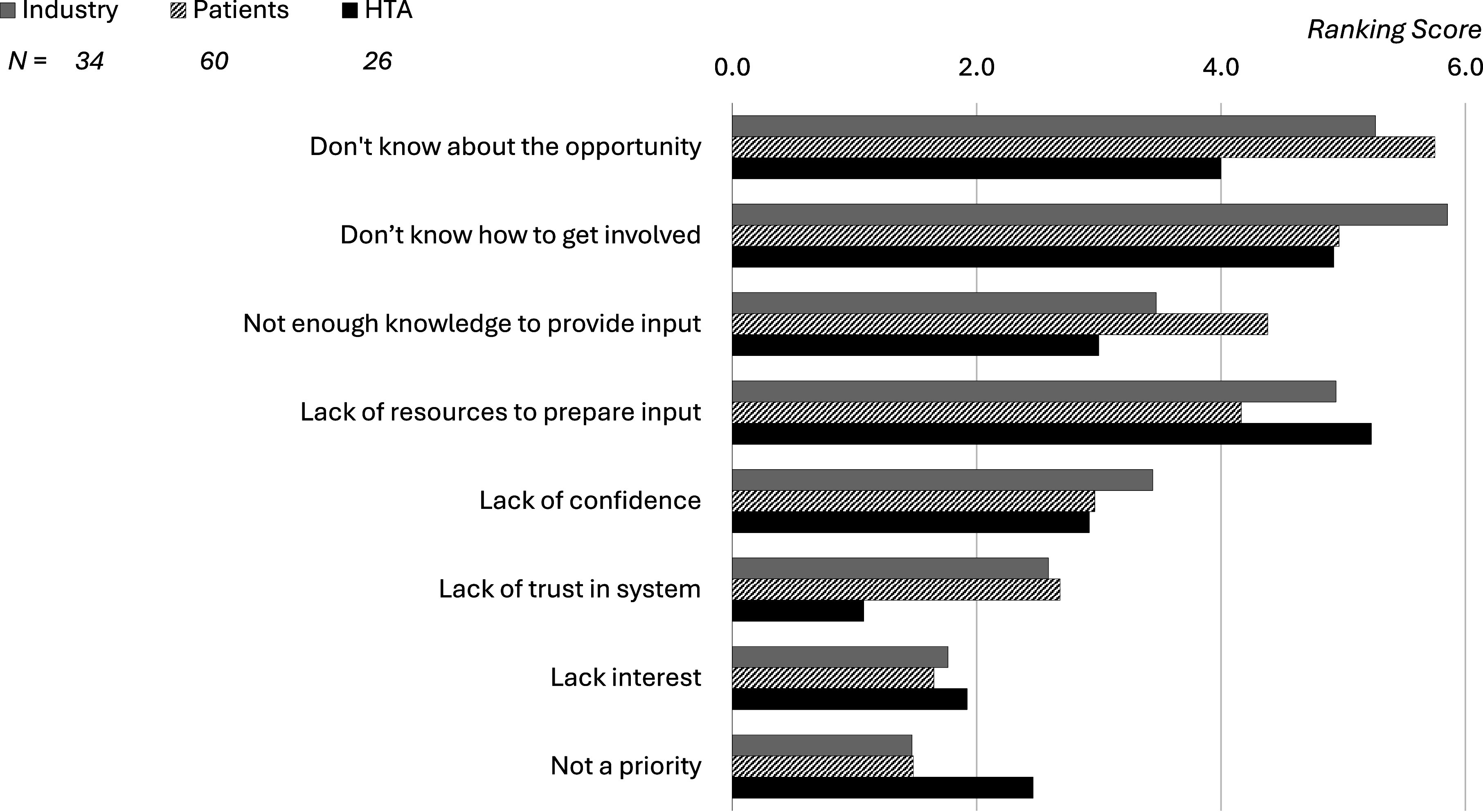
Ranking of reasons that prevent patients from being involved in HTA (Question #B03: “What are the main reasons that prevent patients from being more involved?”).

The four barriers ranked highest (see Figure 2) were that the relevant patients are often not aware of the involvement opportunity, they do not know how to get involved, they lack resources (capacity) to provide input, and they lack the required knowledge (capability). Some patient stakeholders also commented that they do not consider HTA to be a priority.

This ranking was consistent with the items submitted by respondents answering the final survey question, which asked for free-text descriptions of barriers. Here, 367 barrier descriptions were submitted by ninety-eight responders. These responses fell into thirty types (codifiers) of potential barriers. The barriers mentioned most frequently were “*Lack of knowledge among patients (about the opportunity to be involved),”*, “*Lack of resources,”* and “*Lack of guidance/training.”*

**Figure 3.**
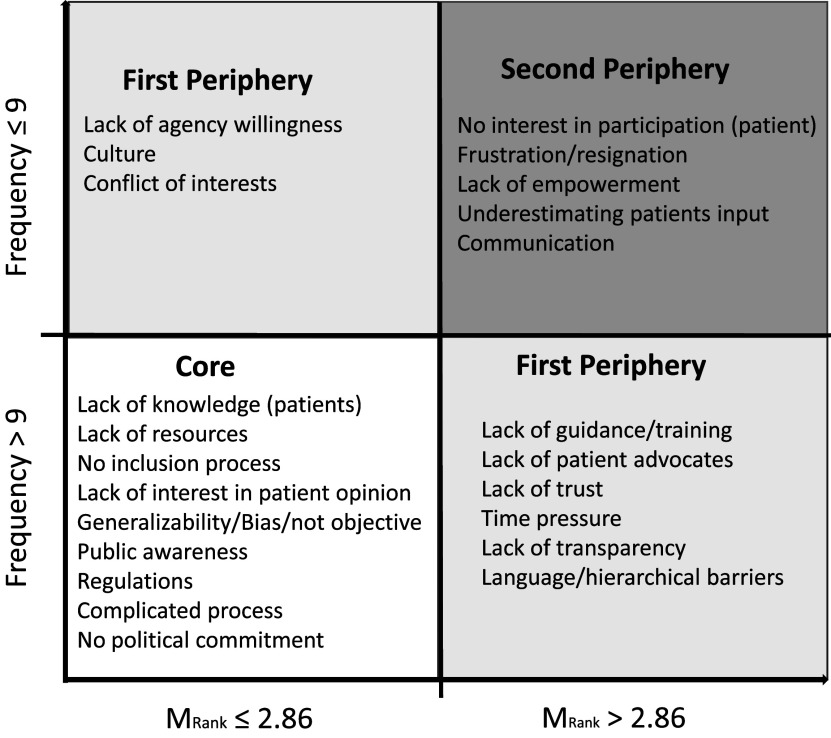
Categories of barriers to patient involvement by frequency and how early they were given.

We conducted a core–periphery analysis (13,14) of the free-text responses for barrier types. Building on social representation theory, this method follows research highlighting that in free associations, the sequencing of responses is important information that can be combined with the frequency of a response – that is barriers that are mentioned earlier in a respondee’s list may have more weight than those entered later. As such, Figure 3 visualizes response categories by those that were named most (defined as appearing ten times or more) and early (defined as a mean rank below 2.68, where “1” would be a category that was named exclusively first and “5” a category that was named exclusively last). Responses that are named both early and often are categorizes as the “core” and defined as the most important response categories. Responses that were named either early or frequently, but not both, are categorized as the “first periphery.” The remaining responses are categorized in the second periphery. While a core–periphery-analysis provides insights into associations made by people and is technically quantitative in nature, it does not provide data on statistical significance or probability.

When asked how patients knew about a specific HTA (Question C-02a), both HTA bodies and patient stakeholders identified e-mailings by the HTA body, alerts from other patient organizations, and announcements on the HTA website as the main channels for informing patients about the opportunity to be involved. HTA practitioners also referred to healthcare providers, industry, or word of mouth as potential channels to publicize the call for participation.

Social media was identified as an additional channel by patients, and several patients specified that they proactively searched (“I regularly look out …”) for such information or were informed by somebody else from their social network.

### Options for strengthening patient involvement in HTA

Three survey questions (B04, B04b, E06) targeted to different responder types explored how patient involvement in HTA could be strengthened.

**Figure 4.**
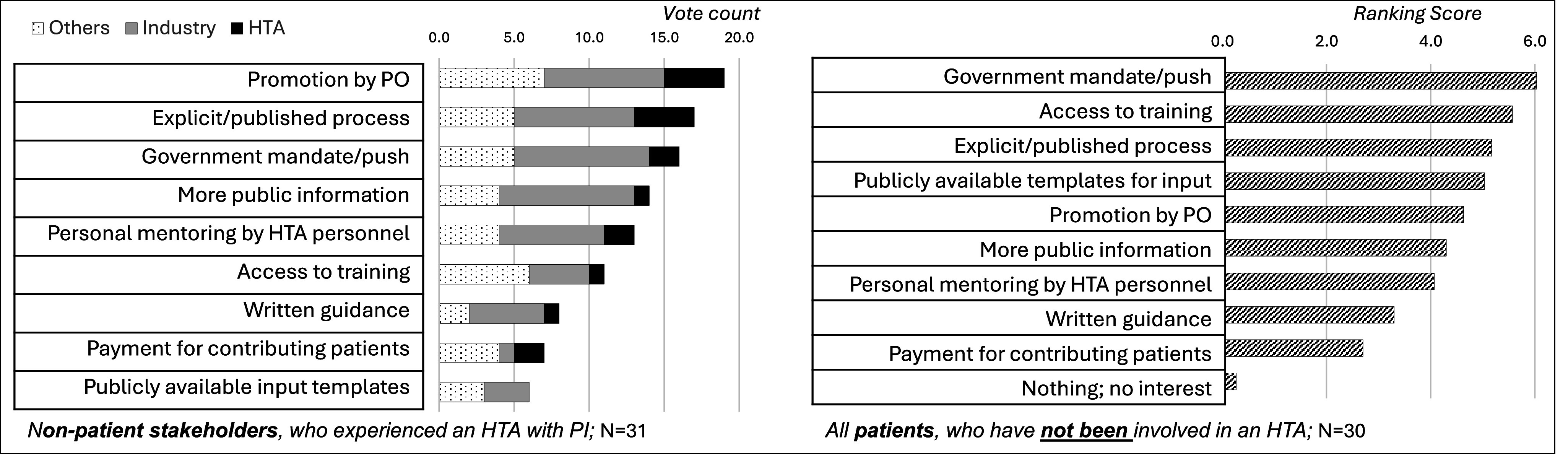
The question (# B04) “What helps to strengthen PI in HTA” was asked to non-patient stakeholders who had experienced patient involvement in HTA (see left graphic) and the question (# B04b) “What would motivate you to become involved?” to those patients who reported not having experienced patient involvement (see right graphic). Abbreviations: PI = Patient Involvement, PO = Patient Organizations, HTA = Health Technology Assessment.

Nonpatient stakeholders with experience of patient involvement in HTA (B04; left graphic in Figure 4) selected “*promotion by patient organizations,”* “*explicit, published processes,”* and a “*government mandate for involving patients in HTA”* (i.e., mandating patient involvement through legislation) as the three most important measures for strengthening patient involvement in HTA.

Patient stakeholders who had not yet been involved in an HTA ranked a “*government mandate for patient involvement,”* followed by “*access to training”* and “*explicit published processes”* as important measures to strengthen patient involvement in HTA (B04b; right graphic in Figure 4).

A final question “*Which measures best strengthen patient involvement in HTA?”* (Question E06) posted to all responders confirmed the previously identified top items as “*Explicit, well-defined processes,”* “*Mandate from government for patient involvement,”* and “*Access to external training*” (*Results not shown*). Here, HTA respondents put more emphasis on processes, training by the HTA body, and formal patient stakeholder submission templates.

### Low level of satisfaction of patient stakeholders with the information they received

**Figure 5.**
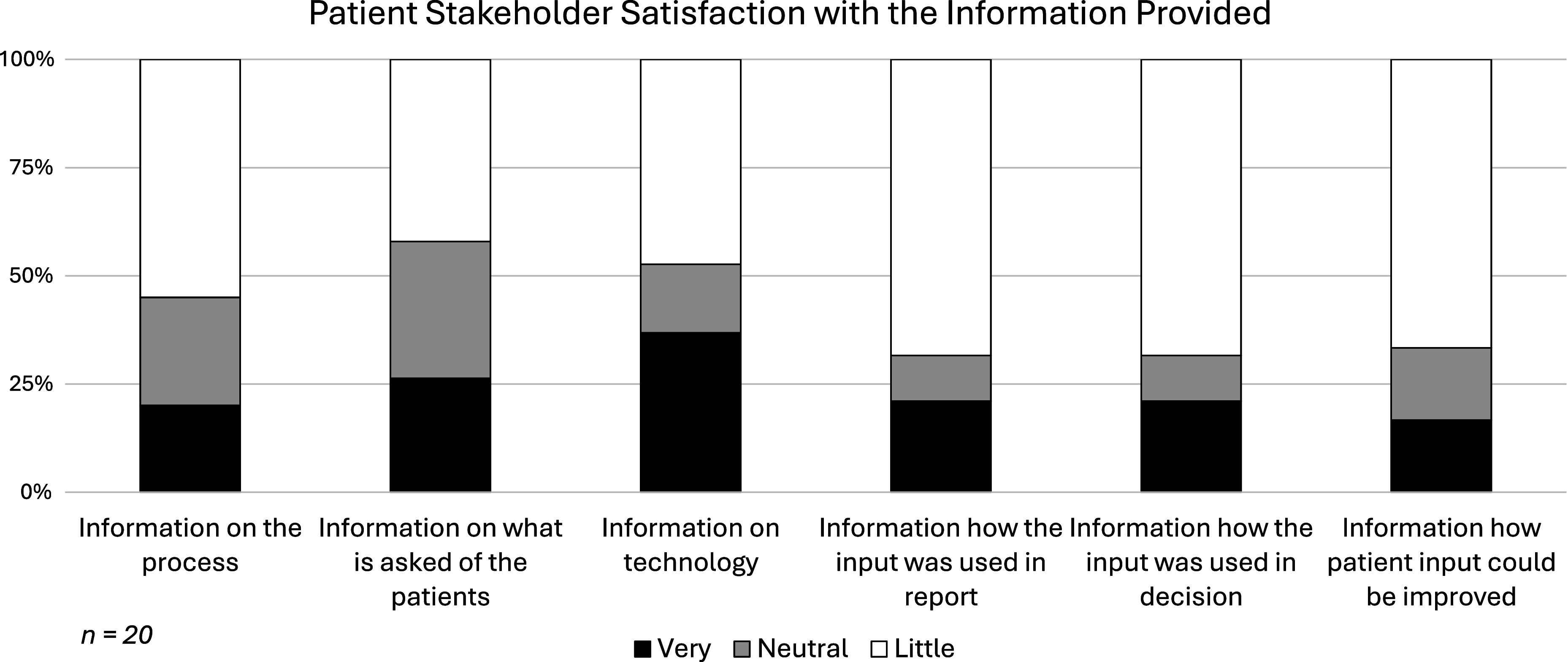
Responses to the question “How satisfied were you with the different types of information provided that explains the patient involvement process and results?” (Question # E03; only to patients who had been involved in HTA; n = 20).

Giving relevant input to HTA requires that appropriate information is given to the stakeholders who are asked for their input. Therefore, those patients who had been involved in HTA were asked for their satisfaction with different aspects of information related to the HTA process they experienced (Question E03 “*How satisfied were you with the different types of information provided that explains the patient involvement process and results*?”). The responses as shown in Figure 5 revealed a generally low satisfaction level: While 37 percent were very satisfied with the technical information given to them, fewer were satisfied with the information on the process (20 percent), regarding what was asked from them (26 percent), with the explanations on how the input was used in the report (21 percent) or the HTA recommendation (21 percent), or regarding how future input could be improved (17 percent).

## Discussion

A survey to explore experiences with patient involvement in HTA across Europe was disseminated broadly through patient organizations, within the HTA community and HTAi membership. The results indicate that involving patients in HTA is perceived not only as having high value across participating stakeholders but also that barriers remain that need to be addressed systematically.

### Overwhelming support for patient involvement in HTA

The vast majority of respondents indicated that patients should be involved in most HTAs. When asked why patients should be involved, “*Patients have insights and information no other stakeholder has”* and “*To inform HTA which evidence is most patient-relevant”* were ranked as the most important reasons without any systematic difference between the responder types.

These responses correlate well with the 2014 HTAi PCIG Values and Quality Standards (5). However, the values of *fairness*, *legitimacy of the decision*, *equity*, and *capacity building* contained in the PCIG Values and Quality Standards were ranked with a lower importance in our survey. It should, however, be noted that the survey required a ranking of the responses; therefore, the results are not directly comparable or contradictive to those gained through the Delphi process in 2014.

### Barriers to patient involvement in HTA

In practice, patients are not always involved in HTA even in those countries where patient involvement in HTA is supported, encouraged or mandatory (10,12). The barriers ranked highest in the survey can be summarized as a lack of knowledge about the opportunity to be involved, how to be involved, as well as lack of knowledge about HTA. In addition, becoming involved in HTA often carries an immense opportunity cost to patients or patient organizations (the time and resources spent are taken away from their other lines of activities). In addition to the time required for the actual involvement event, they need to invest time in preparation, collection of information for their contributions, acquiring the base knowledge of HTA and the technologies in question, and monitoring the official calls for involvement from HTA bodies (15).

To many patient stakeholders, the process is perceived as nontransparent as shown by the low level of satisfaction with the information given to them by the HTA bodies and, most importantly, the perceived lack of feedback on how patient input was used in the report or the recommendation. Hence, as reflected by some patient stakeholder responses, they may decide to avoid HTA involvement in order to concentrate on their core patient support and advocacy activities.

The final question related to barriers allowed free-text responses and revealed more detail. The thirty types of barriers that were extracted from the 367 responses can be attributed to different domains.

*Patient- or patient organization-related barriers* include the lack of knowledge, lack of available or willing patient advocates, resignation (belief that patient input has no impact), or conflicts of interests.

Another group of barriers relates to the *HTA organizations and processes.* These include, for example, technical and complicated processes, perception of patients as not capable, lack of processes, lack of transparency in the involvement, or lack of resources and time.

In addition, *cultural or contextual barriers* for patient involvement may exist such as a general lack of a participatory culture, low level of people empowerment, lack of political commitment, or a restrictive legislative setting.

Another barrier domain, constricted communication, includes items such as fragmented, unidirectional, or selective communication; lack of trust, language or hierarchical barriers; and lack of measurement, feedback, and improvement that prevent learning and progress and likely lead to frustration and resignation.

### Strengthening patient involvement in HTA

Across the different participating stakeholders, high expectations for strengthening patient involvement in HTA were set in the areas of “*process transparency,”* “*collaboration between HTA agencies and patient organizations (e.g., through ‘promotion by patient organizations’),”* and “*access to training and information.”* However, a recommendation or even mandate from governmental policies was highlighted as one of the most promising interventions. Such governmental intervention would strongly depend on the political and cultural context in each country. However, with increasing harmonization of HTA across Europe, a leadership role may be taken by the European Union (EU) Commission through the HTA Regulation that mandates stakeholder involvement in the EU-level clinical assessments starting in January 2025 (16).

#### Training for patient involvement in HTA

Regarding training to improve the preparedness of all parties in patient involvement, patient stakeholders indicated a preference for independent training sources in contrast to HTA respondees who prioritized training from the HTA body itself.

Training options for patients on HTA and their potential involvement opportunities are still limited. Some agencies (e.g., NICE in England) offer a variety of training options (e.g., regular base training, information that is published on the agency’s website) or individual support (e.g., individual coaching through HTA personnel). In addition, there are independent training programs existing or in development in Europe, which are specifically targeted to patients who want to become more engaged in medicines R&D or in HTA (e.g., by EUPATI (17) or some patient organizations). New training initiatives enabled through EU funding and focused on stakeholder involvement in the EU-level Joint Clinical Assessments or Scientific Consultations are scheduled to be completed by 2024/2025 (18,19). Further evaluation will be needed to assess the effectiveness of these new initiatives in overcoming the barriers that were identified in the survey. Their effectiveness in improving the capabilities and willingness of patient stakeholders to be involved remains to be seen. Key questions need to be answered including; What level of knowledge should patient stakeholders have about HTA in order to be able to contribute effectively? What level of knowledge is required for patients to participate in deliberative processes or in decisions on broader public health? Which are the most effective means to convey such knowledge? Analyzing what has worked well in the past (and what has not) should help inform the development of new training formats.

#### Collaboration between HTA and patient organizations

The survey responses indicated that working through patient organizations as a mediator to gain patient input was another important measure. The membership and networks of patient organizations could be used to facilitate a wider outreach to relevant patient populations. Patient organizations could play a double role by, firstly, interpreting and conveying the information from the HTA bodies in ways that make it more digestible to their members and the wider patient population and, secondly, supplying appropriate patient profiles and case examples for involvement in HTA processes. EPF and other patient organizations have increasingly raised awareness about HTA and the value of patient involvement, promoted the need for such involvement, or directly offered educational programs.

Lack of access to patient advocates or representatives was mentioned as one important barrier perceived by HTA organizations. Closer collaboration with patient organizations and more active outreach may help to improve the awareness of the opportunities for involvement.

An example for a multilevel approach to identifying and contacting relevant patient representatives includes seventeen approaches listed by the former Canada’s Drug and Health Technology Agency (now known as Canada’s Drug Agency) (20). This includes approaches through collaboration with patient, caregiver, or consumer organizations, the active and passive use of social media, or longer-term relationship management methods. This set of approaches may serve to prompt others trying to set up a broader multi-channel outreach to potential users of health technologies.

#### Need for information and good practices

The survey responses strongly indicate that improved transparency of the processes for patient involvement in HTA could contribute to the willingness of patient stakeholders to be involved. While some HTA bodies in Europe have defined clear processes and frameworks, most have not, and these HTA bodies adopt methods for patient involvement more incidentally than strategically, often driven by interested individuals taking the lead in the agency (10). In addition, evaluation is not done systematically; therefore, patient involvement often lacks the methodological rigor that is applied to other methods used in HTA (11).

Finally, the survey results highlight that patients who had been involved in HTA processes were strongly dissatisfied in relation to the information they had received (see *Figure 4*). Particularly noteworthy are the low satisfaction rates with (1) information on what was asked of the patient contributors, (2) information on the process, (3) how their input was used in the report or the decision, and (4) information on how they could improve their input in future.

Patient stakeholders must be informed about why they are being asked to participate and given details on what happens to their input. It is not surprising that without this important information, many patient stakeholders are not motivated to devote the time and resources needed to input into HTA. The shortcomings revealed through the survey highlight the pressing need for identifying, developing, and applying good practices in a transparent manner. The results are a clear call to action for HTA bodies to consistently assess their patient involvement practices to identify what works well and what needs to be improved. Without this action, the trust of this essential community will be eroded, and cooperation among all stakeholders will be diminished.

#### Moving toward integrating and improving practices for patient involvement in HTA

Patient involvement can be better anchored in HTA across Europe by addressing the identified barriers in collaboration between HTA practitioners and the other relevant stakeholders. We call on HTA bodies, alongside all the affected stakeholders, to collaborate to address these key barriers and facilitate a better flow of information and dialogue among HTA practitioners and the patient community. Monitoring and analyzing the experiences of those involved in HTA, as well as the expectations and barriers of those who are reluctant to be involved will be critical to achieving open and broad patient involvement in HTA. Collaboration and co-creation will foster trust, mutual learning, and lead to more robust and sustainable patient involvement practices.

## Limitations

Although multiple stakeholder types, including HTA researchers, patient representatives, and industry representatives, contributed to developing the survey, the core group representing these stakeholders was limited to eight people. To alleviate this limitation, the survey was pilot-tested with patient experts, and their feedback was incorporated.

Despite repeated broad dissemination of the survey through the patient community, the HTA community, and among industry stakeholders, only 95 people had had experiences with patient involvement in HTA drawn from 168 responses that were received from thirty-two European countries. If the survey could be applied more consistently over time, the results could also be analyzed to reveal potential differences among the stakeholder types or countries. Repeated and more systematic application of the survey to the stakeholders (HTA researchers, patients, industry) in the same context or jurisdiction might also reveal information for process quality and improvement.

## Conclusion

Patient involvement in HTA is practiced to varying degrees across European countries. Participants in a European-wide survey indicated that they consider patient involvement in HTA to be important, but shortcomings were identified in the lack of systematic and transparent processes, the appropriate level of information, guidance, and related communication and collaboration. Patient involvement currently lacks both consistency and quality. We call on HTA bodies to implement proven and tested practices for involving patients that work well for the participating patients and improve the quality of HTA in Europe. This includes efforts to enhance the content and flow of information related to patient involvement across the lifecycle of HTA. All stakeholders (policymakers, HTA agencies, patient organizations, and potentially industry) should join forces in creating more consistent, aligned, conditions for patient involvement that enable relevance, fairness, equity, and legitimacy of HTA recommendations and/or decisions while improving the quality and standards of patient involvement in HTA.

## Supporting information

Holtorf et al. supplementary material Holtorf et al. supplementary material

## Supplementary material

The supplementary material for this article can be found at http://doi.org/10.1017/S0266462324004707.
