# Supplementary material for: Stakeholder perspectives on the current status and potential barriers of patient involvement in health technology assessment (HTA) across Europe
Source: Int J Technol Assess Health Care. 2024 Dec 19;40(1):e81. doi: 10.1017/S0266462324004707 (PMC11703633; doi:10.1017/S0266462324004707)
Supplement: Holtorf et al. supplementary material [file S0266462324004707sup001.pdf]

Patient Involvement in HTA in Europe: Stakeholder Experiences

Thank you for your interest in this survey.

We are exploring how patient involvement processes used in Health Technology Assessment (HTA) in Europe are experienced and valued by all participants (representatives of HTA agency, industry, patient advocates, and patients).

We are interested in the experiences of all stakeholders who have participated in HTAs with patient involvement such as HTA practitioners and researchers, patients, industry, academics, administrators, or payers.

The research is conducted as a collaboration between the International Society for Health Technology Assessment (HTAi), the European Patients Forum (EPF), and the European Patients' Academy on Therapeutic Innovation (EUPATI). More detailed information about the research can be found on the website of HTAi (https://htai.org/360-hta-patient-involvement-europe/ (https://htai.org/360-hta-patient-involvement-europe/)).

The full survey has five sections (General, Opinions, Pre-HTA, During HTA, After HTA) and will take approximately 30-45 minutes. The survey can be saved, so that you may respond in several sessions. However, it might be more efficient to respond to all questions in one session.

If you have questions or require any other information, please contact Contact E-Mail for Survey (mailto:htai-pcig@health-os.com?subject=Survey %20%22Stakeholder%20Experiences%20with%20Patient%20Invovlement%20in%20HTA%20in%20Europe%22)

For the purpose of this survey, ***patient involvement in HTA*** *is defined as* ***any process to improve the representation of the patient perspective in HTA, including research into patient aspects (patients' experiences, preferences, perspectives), patient participation in the HTA process, or effective and active collaboration with patients, patient advocates, patient representatives and/or carers along with all other relevant stakeholders in the processes and decisions of HTA****.*

There are 52 questions in this survey.

Introduction

SECTION 1: INTRODUCTORY QUESTIONS

The survey has five sections. In this first section we are interested in knowing a bit more about you and your perspective.

From which perspective do you respond to this questionnaire? *

❗ Choose one of the following answers
❗ If you choose 'Other:' please also specify your choice in the accompanying text field.
Please choose **only one** of the following:

- ◯ HTA practitioner / researcher
- ◯ Patient (expert, organization, advocate, other)
- ◯ Industry
- ◯ Provider (Clinician, specialist, nurse etc.)
- ◯ Academic
- ◯ Other ______

If other, please specify in comment

Which patient category describes your role best?

Only answer this question if the following conditions are met:
Answer was 'Patient (expert, organization, advocate, other)' at question ' [A01]' (From which perspective do you respond to this questionnaire?)

❗ Check all that apply
Please choose **all** that apply:

- ☐ **Individual patient**
- ☐ **Care person / Carer**
- ☐ **Patient advocate**
- ☐ **Patient organisation representative**
- ☐ **Patient expert**

The term “patient” is often used as a general, imprecise term that does not reflect the different types of input and experience required from patients, patient advocates and patient organisations in different collaborative processes.
In order to clarify terminology for potential roles of patient interaction presented in this and the other EUPATI guidance documents, we use the term “patient” which covers the following definitions:

- “Individual Patients” are persons with personal experience of living with a disease. They may or may not have technical knowledge in Research and Development or regulatory processes, but their main role is to contribute with their subjective disease and treatment experience.
- “Carers” are persons supporting individual patients such as family members as well as paid or volunteer helpers.
- “Patient Advocates” are persons who have the insight and experience in supporting a larger population of patients living with a specific disease. They may or may not be affiliated with an organisation.
- “Patient Organisation Representatives” are persons who are mandated to represent and express the collective views of a patient organisation on a specific issue or disease area.
- “Patient Experts”, in addition to disease-specific expertise, have the technical knowledge in R&D and/or regulatory affairs through training or experience, for example EUPATI Fellows who have been trained by EUPATI on the full spectrum of medicines R&D.

EUPATI: Guidance for patient involvement in industry-led medicines R&D (https://toolbox.eupati.eu/resources/guidance-for-patient-involvement-in-industry-led-medicines-rd/)

For the purpose of this survey, we are using the following definitions for 'Health Technology' and 'Health Technology Assessment'. **Do you agree with these definitions?**

A ***Health Technology*** *is an intervention developed to prevent, diagnose, or treat medical conditions; promote health; provide rehabilitation; or organize healthcare delivery. The intervention can be a test, device, medicine, vaccine, procedure, program, or system.*

Health Technology Assessment (HTA) *is a multidisciplinary process that uses explicit methods to determine the value of a health technology at different points in its lifecycle. The purpose is to inform decision-making in order to promote an equitable, efficient, and high-quality health system. (Source: HTAi Definition (https://htaglossary.net/health-technology-assessment))*

*

Please choose **only one** of the following:

- ◯ Yes
- ◯ No

Please, briefly explain why you did not agree to the definitions of HT or HTA. (Max 600 characters)

Only answer this question if the following conditions are met:
Answer was 'No' at question ' [A02]' (For the purpose of this survey, we are using the following definitions for 'Health Technology' and 'Health Technology Assessment'. Do you agree with these definitions? --- A Health Technology is an intervention developed to prevent, diagnose, or treat medical conditions; promote health; provide rehabilitation; or organize healthcare delivery. The intervention can be a test, device, medicine, vaccine, procedure, program, or system. --- Health Technology Assessment (HTA) is a multidisciplinary process that uses explicit methods to determine the value of a health technology at different points in its lifecycle. The purpose is to inform decision-making in order to promote an equitable, efficient, and high-quality health system. (Source: HTAi Definition))

Please write your answer here:

Below are several possible reasons why HTA is performed. Please select and rank the ones which you find most important (maximum 5).

❗ All your answers must be different and you must rank in order.

❗ Please select from 1 to 5 answers.

Please number each box in order of preference from 1 to 9

Please choose at least 1 items.

Please choose no more than 5 items.

☐ Understand the value of new treatments

☐ Allow access to treatments that have demonstrated value

☐ Ensure that treatments are affordable

☐ Ensure evidence-based allocation of healthcare resources

☐ Protect healthcare system from treatments that are less effective

☐ Support evidence-based decisions on the access to treatments

☐ Equity for users of healthcare products and services

☐ Save cost in healthcare

☐ Rationalize healthcare decisions

Have you participated in/ contributed to health technology assessment with **Patient Involvement**?

*

Please choose **only one** of the following:

- ◯ Yes
- ◯ No

Which type of Health Technology Assessment with patient involvement have you been involved in?

Only answer this question if the following conditions are met:
Answer was 'Yes' at question ' [A04]' (Have you participated in/ contributed to health technology assessment with Patient Involvement?)

❗ Check all that apply
Please choose **all** that apply:

- ☐ Pharmaceutical Product
- ☐ Medical Device
- ☐ Diagnostic
- ☐ Vaccine
- ☐ Surgical intervention
- ☐ Other: ____________

In which country or region did the HTA take place?

*

Only answer this question if the following conditions are met:
Answer was 'Yes' at question ' [A04]' (Have you participated in/ contributed to health technology assessment with Patient Involvement?)

❗ Choose one of the following answers
❗ If you choose 'Other:' please also specify your choice in the accompanying text field.
Please choose **only one** of the following:

- ◯ Albania
- ◯ Andorra
- ◯ Armenia
- ◯ Austria
- ◯ Azerbaijan
- ◯ Belarus
- ◯ Belgium
- ◯ Bosnia and Herzegovina
- ◯ Bulgaria
- ◯ Croatia
- ◯ Cyprus
- ◯ Czechia
- ◯ Denmark
- ◯ Estonia
- ◯ Finland
- ◯ France
- ◯ Georgia
- ◯ Germany
- ◯ Greece
- ◯ Hungary
- ◯ Iceland
- ◯ Ireland

- ◯ Italy
- ◯ Kazakhstan
- ◯ Kosovo
- ◯ Latvia
- ◯ Liechtenstein
- ◯ Lithuania
- ◯ Luxembourg
- ◯ Malta
- ◯ Moldova
- ◯ Monaco
- ◯ Montenegro
- ◯ Netherlands
- ◯ North Macedonia
- ◯ Norway
- ◯ Poland
- ◯ Portugal
- ◯ Romania
- ◯ Russia
- ◯ San Marino
- ◯ Serbia
- ◯ Slovakia
- ◯ Slovenia
- ◯ Spain
- ◯ Sweden
- ◯ Switzerland
- ◯ Turkey
- ◯ Ukraine
- ◯ United Kingdom (UK)
- ◯ Vatican City (Holy See)
- ◯ Other ______

To which country do your responses relate? *

Only answer this question if the following conditions are met:
((A04.NAOK (/index.php?r=questionAdministration/view/surveyid/202211/gid/108/qid/5130) == 'N'))

❗ Choose one of the following answers
❗ If you choose 'Other:' please also specify your choice in the accompanying text field.
Please choose **only one** of the following:

- ◯ Albania
- ◯ Andorra
- ◯ Armenia
- ◯ Austria
- ◯ Azerbaijan
- ◯ Belarus
- ◯ Belgium
- ◯ Bosnia and Herzegovina
- ◯ Bulgaria
- ◯ Croatia
- ◯ Cyprus
- ◯ Czechia
- ◯ Denmark
- ◯ Estonia
- ◯ Finland
- ◯ France
- ◯ Georgia
- ◯ Germany
- ◯ Greece
- ◯ Hungary
- ◯ Iceland
- ◯ Ireland
- ◯ Italy

- Kazakhstan
- Kosovo
- Latvia
- Liechtenstein
- Lithuania
- Luxembourg
- Malta
- Moldova
- Monaco
- Montenegro
- Netherlands
- North Macedonia
- Norway
- Poland
- Portugal
- Romania
- Russia
- San Marino
- Serbia
- Slovakia
- Slovenia
- Spain
- Sweden
- Switzerland
- Turkey
- Ukraine
- United Kingdom (UK)
- Vatican City (Holy See)
- General - no specific HTA agency or country
- Other

Opinions

SECTION 2: QUESTIONS RELATING TO OPINIONS ABOUT PATIENT INVOLVEMENT IN HTA

In this section of the survey, we would like to understand your expectations towards patient involvement in HTA.

Please remember the definitions:

Patient Involvement and **Health Technology Assessment (HTA)**

In your opinion, should patients be involved in Health Technology Assessment?
*

Choose one of the following answers

Please choose **only one** of the following:

- Always
- In most cases
- In some cases
- Never

Why should patients (patient organizations) be involved in Health Technology Assessment?

Please select and rank the reasons you find most important.

*

Only answer this question if the following conditions are met:
Answer was 'In most cases ' *or* 'In some cases ' *or* 'Always ' at question ' [B01]' (In your opinion, should patients be involved in Health Technology Assessment?)

❗ Please select from 1 to 8 answers.
Please number each box in order of preference from 1 to 8
Please choose at least 1 items.

☐ Patients have insights and information no other stakeholder has

☐ Fairness

☐ Improved quality of information

☐ Legitimacy of the decision

☐ Transparency of decisions

☐ Increase trust in healthcare system decisions

☐ To inform HTA which evidence is most patient-relevant

☐ To respond to specific patient- or carer-related questions

Click on the items you find most important and push them into the box on the right side.
Rank them by importance from top to bottom.

Why should patients not be involved in Health Technology Assessment? *

Only answer this question if the following conditions are met:
Answer was 'Never' *or* 'In some cases ' at question ' [B01]' (In your opinion, should patients be involved in Health Technology Assessment?)

Please write your answer here:

Please give a short answer. If there are several reasons, please separate by comma.

In your opinion, what are the main reasons that prevent patients from being more involved?

Please select and rank the reasons you find most important.

*

❗ Please select from 1 to 8 answers.
Please number each box in order of preference from 1 to 8
Please choose at least 1 items.

- [ ] Lack of resources to prepare input
- [ ] Not a priority
- [ ] Not enough knowledge to provide input
- [ ] Don’t know how to get involved
- [ ] Lack of trust in system
- [ ] Lack interest
- [ ] Lack of confidence
- [ ] Don't know about the opportunity

Click on the items you find most important and push them into the box on the right side. Rank them by importance from top to bottom.

What would help to strengthen Patient Involvement in Health Technology Assessment in your opinion? *

Only answer this question if the following conditions are met:
(((is_empty(A01.NAOK (/index.php?r=questionAdministration/view/surveyid/202211/gid/108/qid/5127)) || (A01.NAOK (/index.php?r=questionAdministration/view/surveyid/202211/gid/108/qid/5127) != 2))) and (A04.NAOK (/index.php?r=questionAdministration/view/surveyid/202211/gid/108/qid/5130) == 'N'))

❗ Check all that apply

❗ Please select from 1 to 5 answers.

Please choose **all** that apply:

- ☐ Mandate from government for patient involvement
- ☐ Explicit (well-defined, structured) process
- ☐ Access to training
- ☐ Written Guidance
- ☐ Publicly available templates for input
- ☐ More public information about it
- ☐ Promotion by Patient organisations
- ☐ Payment of the contributing Patients
- ☐ Personal mentoring through HTA personnel
- ☐ Other: ____________

What would motivate and help you to try to become involved in Health Technology Assessment?

Please select and rank the reasons you find most important.

*

Only answer this question if the following conditions are met:
(((! is_empty(A01.NAOK (/index.php?r=questionAdministration/view/surveyid/202211/gid/108/qid/5127)) && (A01.NAOK (/index.php?r=questionAdministration/view/surveyid/202211/gid/108/qid/5127) == 2))) and (A04.NAOK (/index.php?r=questionAdministration/view/surveyid/202211/gid/108/qid/5130) == 'N'))

❗ All your answers must be different and you must rank in order.

❗ Please select from 1 to 10 answers.

Please number each box in order of preference from 1 to 10

Please choose at least 1 items.

☐ Push from government for patient involvement

☐ Well published process

☐ Access to training

☐ Written Guidance

☐ Publicly available templates for input

☐ More public information about it

☐ Promotion by Patient organisations

☐ Payment for my time

☐ Personal mentoring through HTA personnel

☐ I am not interested

Click on the items you find most important and push them into the box on the right side.

Rank them by importance from top to bottom.

HTA requires the analysis of different types of evidence. Please rank (top to bottom) those 5 evidence types that reflect the patient experience beyond clinical evidence which you find most important.

*

All your answers must be different and you must rank in order.

Please select at most 5 answers

Please number each box in order of preference from 1 to 8

Please choose no more than 5 items.

[ ] **Patient reported outcomes**

[ ] **Health related Quality of Life studies**

[ ] **Written patient experiences**

[ ] **Oral testimonies**

[ ] **Patient input**

[ ] **Patient surveys**

[ ] **Social media research / listening studies**

[ ] **Patient preference evidence**

Click on the items you find most important and push them into the box on the right side.

Rank them by importance from top to bottom.

Pre-HTA

SECTION 3: QUESTIONS RELATING TO THE PREPARATION FOR THE HTA

This section is only directed to respondents who have participated in an HTA with patient involvement.

Please, think about a specific experience of HTA with Patient Involvement when answering the subsequent questions.

In this section, we are trying to understand considerations in the preparation of the HTA.

Please remember the definitions:

Patient Involvement and **Health Technology Assessment (HTA)**

How did you find out about the opportunity to be involved?

Only answer this question if the following conditions are met:
(((! is_empty(A01.NAOK (/index.php?r=questionAdministration/view/surveyid/202211/gid/108/qid/5127)) && (A01.NAOK (/index.php?r=questionAdministration/view/surveyid/202211/gid/108/qid/5127) == 2))) and (A04.NAOK (/index.php?r=questionAdministration/view/surveyid/202211/gid/108/qid/5130) == 'Y'))

Check all that apply

Please choose **all** that apply:

- ☐ I was informed by the HTA body directly
- ☐ I regularly look out for specific HTA opportunities
- ☐ Through a general search
- ☐ Other: ____________

How were the patients informed about the opportunity to be involved? *

Only answer this question if the following conditions are met:
(((is_empty(A01.NAOK (/index.php?r=questionAdministration/view/surveyid/202211/gid/108/qid/5127)) || (A01.NAOK (/index.php?r=questionAdministration/view/surveyid/202211/gid/108/qid/5127) != 2))) and (A04.NAOK (/index.php?r=questionAdministration/view/surveyid/202211/gid/108/qid/5130) == 'Y'))

❗ Check all that apply

Please choose **all** that apply:

- [ ] Internet (news and forums)
- [ ] HTA web-site
- [ ] Mailing by HTA body
- [ ] Patient organisations
- [ ] Doctors, Providers
- [ ] Industry
- [ ] Word of mouth
- [ ] Other: ____________

Through which channel(s) were you informed about the opportunity to be involved? *

Only answer this question if the following conditions are met:
(((! is_empty(A01.NAOK (/index.php?r=questionAdministration/view/surveyid/202211/gid/108/qid/5127)) && (A01.NAOK (/index.php?r=questionAdministration/view/surveyid/202211/gid/108/qid/5127) == 2))) and (A04.NAOK (/index.php?r=questionAdministration/view/surveyid/202211/gid/108/qid/5130) == 'Y'))

❗ Check all that apply

Please choose **all** that apply:

- [ ] Internet (news and forums)
- [ ] HTA web-site
- [ ] Mailing by HTA body
- [ ] Patient organisations
- [ ] Doctors, Providers
- [ ] Industry
- [ ] Word of mouth
- [ ] Other: ____________

Which requirements needed to be met for patient organisations to be involved in **this HTA**?

(If no patient organsations were invovled, please select this option (in italic letters)) *

Only answer this question if the following conditions are met:
((A04.NAOK (/index.php?r=questionAdministration/view/surveyid/202211/gid/108/qid/5130) == 'Y'))

❗ Check all that apply
Please choose **all** that apply:

- [ ] **Certification** of Patient organization
- [ ] **Registration** of patient organization by HTA agency
- [ ] **Declaration of interests**
- [ ] No / limited funding from industry
- [ ] Members are patients with the specific disease
- [ ] None
- [ ] *No patient organisations were involved*
- [ ] Don't know
- [ ] Other: ____________

Which requirements needed to be met for individual patients to be involved in this HTA?

(If no individual patients were involved, please select this option (in italic letters)) *

Only answer this question if the following conditions are met:
((A04.NAOK (/index.php?r=questionAdministration/view/surveyid/202211/gid/108/qid/5130) == 'Y'))

Check all that apply

Please choose **all** that apply:

- ☐ Member of patient organization
- ☐ **Declaration** of interests
- ☐ No funding received from industry
- ☐ Having the disease experience
- ☐ Qualification (e.g. training on HTA)
- ☐ none
- ☐ *No individual patients were involved*
- ☐ Don't know
- ☐ Other: ____________

Did you know what the objectives of patient involvement were in **the HTA** you have participated in? *

Only answer this question if the following conditions are met:
((A04.NAOK (/index.php?r=questionAdministration/view/surveyid/202211/gid/108/qid/5130) == 'Y'))

Please choose **only one** of the following:

- ◯ Yes
- ◯ No

How did you know about the objectives?

Only answer this question if the following conditions are met:
Answer was 'Yes' at question ' [A04]' (Have you participated in/ contributed to health technology assessment with Patient Involvement?) *and* Answer was 'Yes' at question ' [C05]' (Did you know what the objectives of patient involvement were in the HTA you have participated in?)

❗ Check all that apply
Please choose **all** that apply:

- [ ] Objectives of Patient Involvement are defined in guidance
- [ ] Explained to me in person
- [ ] Briefing session
- [ ] Objectives / Expectations are explained on Agency website
- [ ] Other: ______

Was there a published or documented process for Patient Involvement in **the HTA** you participated in? *

Only answer this question if the following conditions are met:
((A04.NAOK (/index.php?r=questionAdministration/view/surveyid/202211/gid/108/qid/5130) == 'Y'))

❗ Check all that apply
❗ Please select at most one answer
Please choose **all** that apply:

- [ ] Yes
- [ ] No
- [ ] Don't know

How was the process created?

Only answer this question if the following conditions are met:
Answer was 'Yes' at question ' [A04]' (Have you participated in/ contributed to health technology assessment with Patient Involvement?) *and* Answer was 'Yes' at question ' [C07]' (Was there a published or documented process for Patient Involvement in the HTA you participated in?)

❗ Check all that apply
Please choose **all** that apply:

- ☐ By HTA agency experts
- ☐ Co-creation with patient organisation(s)
- ☐ Followed HTAi guidance
- ☐ External consultants
- ☐ Don’t know
- ☐ Other: ____________

What was the role of patients (or patient organisations) in the HTA?

Only answer this question if the following conditions are met:
((A04.NAOK (/index.php?r=questionAdministration/view/surveyid/202211/gid/108/qid/5130) == 'Y'))

❗ Check all that apply
Please choose **all** that apply:

- ☐ Equal contributor across the whole process
- ☐ Representation of patient perspective in a specific event (e.g. hearing, focus group, committee meeting)
- ☐ Information broker: Collect & submit patient input info
- ☐ Advise on evaluation process and content
- ☐ not sure
- ☐ Other: ____________

Which resources were provided to support patient involvement? *

Only answer this question if the following conditions are met:
((A04.NAOK (/index.php?r=questionAdministration/view/surveyid/202211/gid/108/qid/5130) == 'Y'))

Please choose the appropriate response for each item:

	Yes	Uncertain	No
**General training related to HTA for patients**	○	○	○
**Specific training on the processes used by this HTA body**	○	○	○
**Training on Patient Involvement for HTA researchers**	○	○	○
**Written guidance for Patient Involvement**	○	○	○
**Dedicated support function for Patient Involvement**	○	○	○
**General written guidance**	○	○	○
**Financial support for participants**	○	○	○
**Templates for input**	○	○	○
**Training for patient participants in public speaking**	○	○	○
**Other (if selected, specify in next question)**	○	○	○

What other support was given?

Only answer this question if the following conditions are met:
Answer was 'Yes' at question ' [A04]' (Have you participated in/ contributed to health technology assessment with Patient Involvement?) *and* Answer was 'Yes' at question ' [C10]' (Which resources were provided to support patient involvement? (Other (if selected, specify in next question)))

Please write your answer here:

What information did the patients receive for **this particular HTA**? *

Only answer this question if the following conditions are met:
((A04.NAOK (/index.php?r=questionAdministration/view/surveyid/202211/gid/108/qid/5130) == 'Y'))

Check all that apply
Please choose **all** that apply:

- [ ] Summary in lay language **(SIP)**
- [ ] Full evidence package for product
- [ ] Selected evidence for product
- [ ] Minimal product related evidence
- [ ] Information through individual briefings
- [ ] Other:

HTA

SECTION 4: QUESTIONS RELATING TO THE HEALTH TECHNOLOGY ASSESSMENT

This section is only directed to respondents who have participated in an HTA with patient involvement.

In this section, we are trying to understand all considerations during the HTA.

Please, think about a specific experience of HTA with Patient Involvement when answering the subsequent questions.

Please remember the definitions:

Patient Involvement and **Health Technology Assessment (HTA)**

At what stages of the HTA are patients involved?

(*individual patients* or *carers*, *patient experts*, *patient organisation representatives* or *patient advocates*)

❗ Check all that apply

Please choose **all** that apply:

- [ ] Scoping
- [ ] Literature review
- [ ] Evidence generation / Research
- [ ] Data analysis
- [ ] Evidence assessment
- [ ] Report
- [ ] Appraisal committee
- [ ] Hearing
- [ ] Consultation
- [ ] Disinvestment
- [ ] Pricing
- [ ] Reimbursement / Coverage

Please indicate for each process whether patients were involved according to your knowledge.

What type of patients are involved in {(D01_1.question)} ?

Only answer this question if the following conditions are met:
((D01_1.NAOK (/index.php?r=questionAdministration/view/surveyid/202211/gid/111/qid/5167) == "Y"))

Check all that apply

Please choose **all** that apply:

- [ ] Individual patient
- [ ] Care person / Carer
- [ ] Patient advocate
- [ ] Patient organisation representative
- [ ] Patient expert
- [ ] Other: ____________

What type of patients are involved in {(D01_2.question)} ?

Only answer this question if the following conditions are met:
((D01_2.NAOK (/index.php?r=questionAdministration/view/surveyid/202211/gid/111/qid/5167) == "Y"))

Check all that apply

Please choose **all** that apply:

- [ ] Individual patient
- [ ] Care person / Carer
- [ ] Patient advocate
- [ ] Patient organisation representative
- [ ] Patient expert
- [ ] Other: ____________

What type of patients are involved in {(D01_3.question)} ?

Only answer this question if the following conditions are met:
((D01_3.NAOK (/index.php?r=questionAdministration/view/surveyid/202211/gid/111/qid/5167) == "Y"))

❶ Check all that apply
Please choose **all** that apply:

- [ ] Individual patient
- [ ] Care person / Carer
- [ ] Patient advocate
- [ ] Patient organisation representative
- [ ] Patient expert
- [ ] Other: ______

What type of patients are involved in {(D01_4.question)} ?

Only answer this question if the following conditions are met:
((D01_4.NAOK (/index.php?r=questionAdministration/view/surveyid/202211/gid/111/qid/5167) == "Y"))

❶ Check all that apply
Please choose **all** that apply:

- [ ] Individual patient
- [ ] Care person / Carer
- [ ] Patient advocate
- [ ] Patient organisation representative
- [ ] Patient expert
- [ ] Other: ______

What type of patients are involved in {(D01_5.question)} ?

Only answer this question if the following conditions are met:
((D01_5.NAOK (/index.php?r=questionAdministration/view/surveyid/202211/gid/111/qid/5167) == "Y"))

❶ Check all that apply

Please choose **all** that apply:

- [ ] Individual patient
- [ ] Care person / Carer
- [ ] Patient advocate
- [ ] Patient organisation representative
- [ ] Patient expert
- [ ] Other: ____________

What type of patients are involved in {(D01_6.question)} ?

Only answer this question if the following conditions are met:
((D01_6.NAOK (/index.php?r=questionAdministration/view/surveyid/202211/gid/111/qid/5167) == "Y"))

❶ Check all that apply

Please choose **all** that apply:

- [ ] Individual patient
- [ ] Care person / Carer
- [ ] Patient advocate
- [ ] Patient organisation representative
- [ ] Patient expert
- [ ] Other: ____________

What type of patients are involved in {(D01_7.question)} ?

Only answer this question if the following conditions are met:
((D01_7.NAOK (/index.php?r=questionAdministration/view/surveyid/202211/gid/111/qid/5167) == "Y"))

❗ Check all that apply
Please choose **all** that apply:

- [ ] Individual patient
- [ ] Care person / Carer
- [ ] Patient advocate
- [ ] Patient organisation representative
- [ ] Patient expert
- [ ] Other: ____________

What type of patients are involved in {(D01_8.question)} ?

Only answer this question if the following conditions are met:
((D01_8.NAOK (/index.php?r=questionAdministration/view/surveyid/202211/gid/111/qid/5167) == "Y"))

❗ Check all that apply
Please choose **all** that apply:

- [ ] Individual patient
- [ ] Care person / Carer
- [ ] Patient advocate
- [ ] Patient organisation representative
- [ ] Patient expert
- [ ] Other: ____________

What type of patients are involved in {(D01_9.question)} ?

Only answer this question if the following conditions are met:
((D01_9.NAOK (/index.php?r=questionAdministration/view/surveyid/202211/gid/111/qid/5167) == "Y"))

❶ Check all that apply
Please choose **all** that apply:

- [ ] Individual patient
- [ ] Care person / Carer
- [ ] Patient advocate
- [ ] Patient organisation representative
- [ ] Patient expert
- [ ] Other: ____________

What type of patients are involved in {(D01_10.question)} ?

Only answer this question if the following conditions are met:
((D01_10.NAOK (/index.php?r=questionAdministration/view/surveyid/202211/gid/111/qid/5167) == "Y"))

❶ Check all that apply
Please choose **all** that apply:

- [ ] Individual patient
- [ ] Care person / Carer
- [ ] Patient advocate
- [ ] Patient organisation representative
- [ ] Patient expert
- [ ] Other: ____________

What type of patients are involved in {(D01_11.question)} ?

Only answer this question if the following conditions are met:
((D01_11.NAOK (/index.php?r=questionAdministration/view/surveyid/202211/gid/111/qid/5167) == "Y"))

❗ Check all that apply
Please choose **all** that apply:

- [ ] Individual patient
- [ ] Care person / Carer
- [ ] Patient advocate
- [ ] Patient organisation representative
- [ ] Patient expert
- [ ] Other: ____________

What type of patients are involved in {(D01_12.question)} ?

Only answer this question if the following conditions are met:
((D01_12.NAOK (/index.php?r=questionAdministration/view/surveyid/202211/gid/111/qid/5167) == "Y"))

❗ Check all that apply
Please choose **all** that apply:

- [ ] Individual patient
- [ ] Care person / Carer
- [ ] Patient advocate
- [ ] Patient organisation representative
- [ ] Patient expert
- [ ] Other: ____________

What was done **<u>in the particular HTA</u>** to improve the representativeness of the patient input? *

Only answer this question if the following conditions are met:
((A04.NAOK (/index.php?r=questionAdministration/view/surveyid/202211/gid/108/qid/5130) == 'Y'))

❗ Check all that apply

Please choose **all** that apply:

- [ ] Evidence generated by patient organisations was considered
- [ ] Surveys of patients were considered
- [ ] An individual patient gave input
- [ ] A panel of patients provided input
- [ ] No specific activity
- [ ] Other: ____________

How did patients give their input?

Only answer this question if the following conditions are met:
Answer was 'Yes' at question ' [A04]' (Have you participated in/ contributed to health technology assessment with Patient Involvement?)

Check all that apply
Please choose **all** that apply:

- [ ] **Testimonies**
- [ ] **Input templates**
- [ ] Participate in multi-stakeholder meeting
- [ ] Written input
- [ ] Interview by HTA researcher
- [ ] Workshops
- [ ] Focus group
- [ ] Consultation (feedback on draft report)
- [ ] **Patient Based Evidence**
- [ ] Other: ____________

Did the HTA report explicitly detail the patient inputs?

Only answer this question if the following conditions are met:
((A04.NAOK (/index.php?r=questionAdministration/view/surveyid/202211/gid/108/qid/5130) == 'Y'))

Check all that apply

Please choose **all** that apply:

- [ ] As part of the main report
- [ ] As an appendix to report
- [ ] In online repository
- [ ] As separate feedback to the patients
- [ ] Only if important to the recommendation
- [ ] Depends on rapporteur
- [ ] Not specifically reported
- [ ] I don't know
- [ ] Other: ____________

Did the HTA report explicitly detail how the patient inputs were considered in the recommendation? *

Only answer this question if the following conditions are met:
((A04.NAOK (/index.php?r=questionAdministration/view/surveyid/202211/gid/108/qid/5130) == 'Y'))

Check all that apply

Please choose **all** that apply:

- [ ] As standard part of report
- [ ] As separate feedback to the patients
- [ ] Only if important to the recommendation
- [ ] Depends on rapporteur
- [ ] Not specifically reported
- [ ] Don't know
- [ ] Other: ____________

Evaluation

FINAL SECTION: EVALUATION, FACILITATORS AND BARRIERS

Please, think about your experience of Patient Involvement in HTA when answering the subsequent questions.

In this final section, we are trying to explore evaluation aspects, learnings and experience.

What aspects of Patient Involvement in HTA do you evaluate in your country or HTA process? *

Only answer this question if the following conditions are met:
Answer was 'HTA practitioner / researcher' at question ' [A01]' (From which perspective do you respond to this questionnaire?)

❗ Check all that apply
Please choose **all** that apply:

- ☐ None
- ☐ Process
- ☐ Satisfaction
- ☐ Impact
- ☐ Don't know
- ☐ Other: ________

Which evaluation mechanisms are applied?

Only answer this question if the following conditions are met:
Answer was 'HTA practitioner / researcher' at question ' [A01]' (From which perspective do you respond to this questionnaire?) *and* Answer was 'Yes' at question ' [A04]' (Have you participated in/ contributed to health technology assessment with Patient Involvement?) *and* Answer was at question ' [E01]' (What aspects of Patient Involvement in HTA do you evaluate in your country or HTA process?) *and* Answer was at question ' [E01]' (What aspects of Patient Involvement in HTA do you evaluate in your country or HTA process?)

Check all that apply
Please choose **all** that apply:

- [ ] Occasional surveys
- [ ] Continuous monitoring throughout the involvement
- [ ] Feedback mechanism built into each assessment
- [ ] Selective interviews
- [ ] Quantitative evaluation (e.g. number of interactions, number of patient inputs, …)
- [ ] Formal consultation processes
- [ ] Don’t know
- [ ] Other: ____________

How satisfied were you with the different types of information provided that explains the patient involvement process and results? *

Only answer this question if the following conditions are met:
(((! is_empty(A01.NAOK (/index.php?r=questionAdministration/view/surveyid/202211/gid/108/qid/5127)) && (A01.NAOK (/index.php?r=questionAdministration/view/surveyid/202211/gid/108/qid/5127) == 2))) and (A04.NAOK (/index.php?r=questionAdministration/view/surveyid/202211/gid/108/qid/5130) == 'Y'))

Please choose the appropriate response for each item:

	Little	Neutral	Very	Didn't apply
**Information on the process**	○	○	○	○
**Information on what is asked of the patients**	○	○	○	○
**Information on technology**	○	○	○	○
**Information how the input was used in report**	○	○	○	○
**Information how the input was used in decision**	○	○	○	○
**Information how patient input could be improved**	○	○	○	○

Were/are the objectives of the patient involvement met in your experience?

Only answer this question if the following conditions are met:
((A04.NAOK (/index.php?r=questionAdministration/view/surveyid/202211/gid/108/qid/5130) == 'Y'))

Please choose the appropriate response for each item:

	NO	Neutral	YES
**Understand the value of new treatments**	○	○	○
**Allow access to treatments that have demonstrated value**	○	○	○
**Ensure that treatments are affordable**	○	○	○
**Ensure evidence-based allocation of healthcare resources**	○	○	○
**Protect healthcare system from treatments that are less effective**	○	○	○
**Support evidence-based decisions on the access to treatments**	○	○	○

Which measures best strengthen Patient Involvement in Health Technology Assessment? *

Only answer this question if the following conditions are met:
((A04.NAOK (/index.php?r=questionAdministration/view/surveyid/202211/gid/108/qid/5130) == 'Y'))

❗ All your answers must be different and you must rank in order.

❗ Please select from 1 to 10 answers.

Please number each box in order of preference from 1 to 10

Please choose at least 1 items.

- [ ] Mandate from government for patient involvement
- [ ] Explicit (well-defined, structured) process
- [ ] Training by HTA agency
- [ ] Access to external training
- [ ] Written Guidance
- [ ] Personal mentor
- [ ] Experience from previous HTAs
- [ ] Templates for input
- [ ] Aligned objectives across stakeholders
- [ ] Feedback mechanisms

In your experience, what are the barriers for patient involvement?

(only one word or very short description possible for a maximum of the five most important barriers) *

❶ Please fill in from 1 to 5 answers.

Thank you for your patience in responding to our survey.

If you interested in receiving information on the outcome of this research, please register your name and E-Mail address under this separate link: http://survey.health-os.com/index.php?r=survey/index&sid=469716&lang=en (http://survey.health-os.com/index.php?r=survey/index&sid=469716&lang=en)

07-09-2022 – 23:59
Please fax your completed survey to: +41-61-3830665
Submit your survey.
Thank you for completing this survey.
